# Use of Economic Evidence When Prioritising Public Health Interventions in Schools: A Qualitative Study with School Staff

**DOI:** 10.3390/ijerph17239077

**Published:** 2020-12-04

**Authors:** Katie Breheny, Emma Frew, Iestyn Williams, Sandra Passmore, Joanna Coast

**Affiliations:** 1 Bristol Medical School, University of Bristol, Bristol BS8 1NU, UK; katie.breheny@bristol.ac.uk; 2 Institute of Applied Health Research, University of Birmingham, Birmingham B15 2TT, UK; e.frew@bham.ac.uk; 3 Health Services Management Centre, School of Social Policy, University of Birmingham, Birmingham B15 2RT, UK; i.p.williams@bham.ac.uk; 4 Services for Education, Birmingham B7 4AX, UK; sandra.passmore@servicesforeducation.co.uk

**Keywords:** schools, economic evaluation, qualitative research, decision making, cost-effectiveness

## Abstract

Schools are an ideal setting to deliver public health interventions, yet there are competing obligations that could limit their implementation. This study aimed to examine the decision making process and explore what evidence informs prioritisation of public health interventions in this setting. Semi-structured interviews were conducted with 14 staff in seven UK schools between November 2017 and March 2018. Participants were recruited from schools participating in The Birmingham Daily Mile trial and comprised leadership staff, teachers, and pastoral staff. Analyses used a constant comparison approach to explore the prioritisation process and schools’ use of economic evidence. Teachers felt that they had little decision making influence in regard to public health interventions, with this falling on leadership staff. Participants perceived tension between delivering academic subjects and public health initiatives and thought proven impact was important to justify the opportunity cost. Evidence did not appear to be routinely used, and participants were unaware of cost-effectiveness analyses, but thought it could be a useful tool. This study shows that schools face challenges in balancing the academic, health, and wellbeing needs of children. There is a need for targeted evidence that includes appropriate costs and outcomes and meets school decision makers’ needs.

## 1. Introduction

As children spend a significant amount of time in school, it provides an ideal environment in which to deliver public health interventions to this population [1]. Public health interventions, however, divert time and resources away from what may be perceived as schools’ main goal of achieving academic attainment [2]. This is despite evidence that health improvement initiatives could have a positive effect on academic performance in secondary school [2,3,4].

Delivering public health interventions in schools is a challenge internationally, due to fragmentation in arrangements for both funding and provision. UK schools are required to offer a “broad and balanced” curriculum through a variety of initiatives including the National Healthy Schools Rating Scheme [5] and a School Fruit and Vegetable Scheme (SFVS), whereby children (aged 4–6 years) receive a fresh portion of fruit or vegetable per day [6]. This has since been updated to also include “relationships and health education”, which is a compulsory requirement for UK primary schools (children aged 4 to 11 years) from September 2020 [7]. Children will be taught the importance of physical and mental wellbeing, including a good diet, an active lifestyle, and how to recognise and/or prevent ill health. In the USA, the Centre for Disease Control and Prevention (CDC) Healthy Schools programme [8] promotes good nutrition, physical activity, and health literacy, although this is not mandatory. Some state education agencies receive funding to support implementation of evidence-based strategies recommended by the programme. Body mass index (BMI) screening in schools has been widely adopted to facilitate population surveillance of childhood obesity and in some cases provide feedback to guardians [9]. In the UK, school nurses are funded by local authorities to administer the National Child Measurement Programme (NCMP) [10]. In the USA, more than 15 states mandate screening, with school nurses largely administering it. Funding is variable, however, with only some states meeting the costs of the programme [9]. Costs otherwise fall on schools to provide the equipment and administrative support. In Australia and the UK, schools are advised to provide two hours of planned physical activity per week [11]. There are a range of public health initiatives that schools can implement at their discretion and using their own funds, one of which is The Daily Mile [12], which has been implemented in over 75 countries [13].

The question of how schools use economic evidence to inform decision making is largely unexplored, although a recent UK study investigating schools’ provision of health promotion found that there was a general interest in evidence, and prioritisation of options was informed by financial considerations [14]. Factors inhibiting provision included budgets and a limited understanding of what was effective. Some staff believed that accessing reliable evidence would itself be costly, whereas others did not have the skills or time to examine the volume of information available. Similarly, Arnold et al. [15] found that funding constraints were a barrier to the implementation of physical activity provision, with academic attainment in literacy and numeracy prioritised. In the USA, a survey of school board members (equivalent of UK governors) found that the two most frequently reported barriers to physical activity were limited budgets and available time [16]. This suggests that there is a need for evidence of cost-effectiveness to assist schools with the prioritisation of public health interventions, and for its availability and accessibility to be considered.

Estimating the cost-effectiveness of public health interventions is challenging [17,18] and “traditional” economic evaluation methods developed in the context of the health service [19] do not translate well to interventions designed to prevent ill health or promote good health. Difficulties posed in the evaluation of public health interventions include identifying both the costs being incurred by the provider, such as the school, and the benefits being observed in the future and within health and other sectors. Factors such as inequalities are also not routinely considered. The National Institute for Health and Care Excellence (NICE) [19] have proposed a broader approach to economic evaluation for public health, which incorporates costs and benefits beyond healthcare, although its suitability for schools’ evidence needs is uncertain. Despite this, there is a broad literature evaluating the cost-effectiveness of school based public health interventions, with many focusing on childhood obesity prevention [20].

This study aimed to examine how public health interventions delivered in UK primary schools are prioritised compared to their competing responsibilities and what consideration is given to evidence, with a focus on economic evidence. Understanding what evidence, if any, is used and whether there is a need for economic evidence can contribute to the development and refinement of methods for evaluating public health interventions within schools and advance the effective dissemination of new and existing research findings.

## 2. Methods

Semi-structured interviews were conducted with teachers, leadership staff (e.g., headteachers, deputy heads, principals, vice-principals),and other staff involved in health and wellbeing activities (e.g., pastoral staff) at schools participating in The Birmingham Daily Mile cluster randomised controlled trial (RCT) [21,22]. The Daily Mile involves children running or walking for 15 min every day. Forty primary schools were randomised to provide either The Daily Mile (intervention) or their usual health and wellbeing activities (control), implementing the intervention for one year. Trial outcomes included BMI, fitness (linear track test), wellbeing (Middle Years Development Instrument (MDI) [23]), quality of life (CHU9D [24]), and academic attainment (teacher reported).

### 2.1. Sample and Recruitment

Eligible participants were those working in schools participating in the intervention arm of The Birmingham Daily Mile trial and able to provide informed consent. This was due to the focus on the implementation of The Daily Mile, although the topic guide allowed for discussion of other public health interventions. It was intended that participants would be recruited and interviewed until saturation of themes around priorities, outcomes, and costs of The Daily Mile had been achieved. A purposive sampling approach was planned, whereby participants would be selected by the researcher based on a variety of criteria for maximum variation [25,26]. Criteria included school characteristics such as number of pupils, eligibility for free school meals, in addition to participant characteristics such as a leadership or teaching role. In order to achieve this sample diversity and saturation, we invited participation from all 20 schools in the intervention arm of the trial.

The staff member with responsibility for coordinating the intervention at each school was first approached by email to participate. Follow-up emails and phone calls were made if no response was received. The invitation contained a participant information sheet detailing the research aim and what participation would involve. A snowballing approach was then used, whereby participants were asked to recommend other individuals within, or working with, the school that they believed would provide a helpful contribution to the study.

### 2.2. Data Collection

Face-to-face interviews were conducted at the participants’ schools; telephone interviews were used where these were the participants’ preference. Locations included offices, staff rooms, and available classrooms. All participants provided informed consent. The interview broadly followed a topic guide that included questions regarding implementation of The Daily Mile, their views on economic evidence, and the prioritisation of health and wellbeing interventions in schools. The Daily Mile is largely considered a physical activity intervention that could form part of a population-wide obesity prevention strategy and lead to wider benefits on pupils’ quality of life and wellbeing, and therefore, the conversation was naturally focused on these types of initiatives. The topic guide was reviewed and refined by members of the project team and adapted as interviews progressed. The interviews were audio-recorded with the participants’ permission.

### 2.3. Data Analysis

Audio data were transcribed verbatim and checked for accuracy. NVivo 12 [27] was used to organise and manage the data. A constant comparative method was used whereby data were repeatedly compared using coding sets and moved around within changing coding sets. New data were compared, initially to previous data and, as analysis progressed, to the properties of emerging themes. Saturation was determined by the absence of any new themes arising in the data. One researcher conducted the initial analysis (KB) and a second reviewed a sample of transcripts to compare themes (JC). Three detailed analytic accounts were developed [28]. These were grouped into: leadership staff; teachers responsible for their own class; other staff (e.g., staff with pastoral roles and teachers delivering physical education (PE) only). These accounts were subsequently drawn together to generate a single account that summarised findings related to the prioritisation of health and wellbeing interventions

Approval for this qualitative study was granted by the University of Birmingham Research Ethics Committee (ERN_17-0171).

## 3. Results

**Figure 1:**
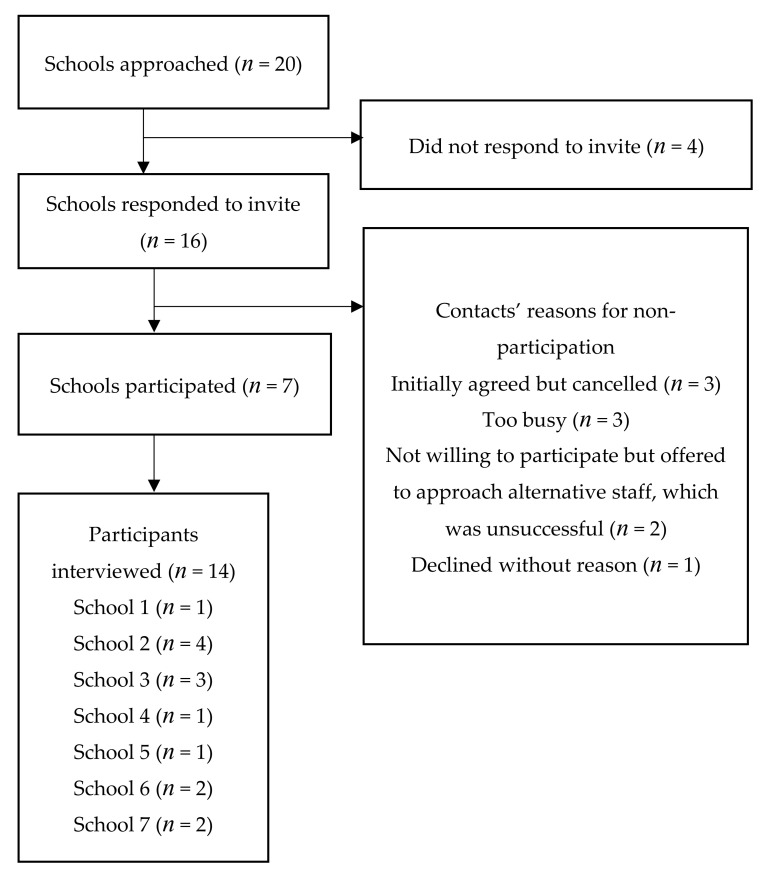
Flow diagram of participant recruitment.

Semi-structured, face-to-face (*n* = 12), or telephone (*n* = 2) interviews were conducted by one individual (KB) and took place between November 2017 and March 2018. Mean interview duration was 28 min. Figure 1 demonstrates the recruitment process and reasons for non-participation. The participants were categorised as leadership staff (*n* = 6), teachers (*n* = 4), and other staff (including safeguarding leads and teachers specialising in PE only) (*n* = 4). Some staff had both leadership and part-time teaching roles. School work experience ranged from 3 to 33 years.

In this section, the findings are described according to the themes that were identified during the analysis. First, the school prioritisation process is described, highlighting how public health initiatives sit alongside schools’ other obligations, then, what and how evidence is used to inform these priorities are explored.

### 3.1. Decision Making Process and Objectives

Participants highlighted that schools are offered or encounter what they considered to be an unfeasible number of public health initiatives. Prioritisation processes were therefore perceived as being necessary. In addition to costs associated with initiatives, limits were necessary because of constraints on time and physical space. When participants were asked who made such decisions, leadership staff were unanimously reported to be the primary decision makers, and this was predominantly seen as being the head teacher (principal). Participants also mentioned that personal factors such as their enthusiasm and personal interest in an initiative seemed to be important when leadership staff were considering implementation. The subsequent success of an initiative was attributed to enthusiastic staff driving it forward. Others involved in the school—such as governors on the school board—were generally seen as less influential in such decisions.

*Schools do tend to get offered an awful lot of things to do, but it’s having someone to implement it, oversee it, get it started, get it finished so we can’t say ‘yes’ to everything, much as we’d like to [ID6, Pastoral role].*

*Well, I’m the head teacher, so normally if there’s an initiative or anything like that and if I’m enthusiastic about it, it normally gets done [ID12, Leader].*

*I’d quite like to go to governors and show them you know that this is what we’re investing in, this is part of our curriculum and this is the impact it’s had [ID9, Leader].*

*I think, personally, it depends on the type of leaders you’ve got in school. It depends on people’s own personal passion. I mean in every school you go in, there probably is somebody who’s into sport and I think if they’re really keen and they go up to the Senior Leaders; have a talk to them about it; show them research about the positives of it, and then I think that’s how you can drive it [ID7, Leader/teacher].*

Public health interventions fit alongside schools’ other responsibilities and mandatory requirements. The primacy of academic attainment was asserted frequently by leadership staff, teachers, and other staff. Many argued that they had to prioritise what they are measured on, which in the UK context is English and Maths outcomes at a school level. It was highlighted that, with targets to meet for these outcomes, health and wellbeing initiatives were inevitably given lower priority. Several participants thought that The Daily Mile could not displace Maths and English lessons but could be substituted for other lessons such as history and art, or for school assemblies. In contrast, it was felt by one leadership staff member that maintaining concentration on Maths or English for long periods of time was unlikely to be productive and simple physical activity breaks, such as The Daily Mile, might be beneficial to learning.

*Schools’ priority has to be what we’re measured on, as it is in all sort of walks of life; be it, hospitals; be it doctors’ surgeries; be it the police. We’ve now all got targets that we’re working towards. If you ask us to go and dedicate a certain amount of time to something that is not directly going to impact on those targets, it’s always going to be hard [ID11, Safeguarding role].*

*If you’ve got two hours of non-stop, you know, Maths and English, you’re going to have that kind of level of concentration that will just dip. I mean nobody can concentrate for that length of time. By having a bit of a burst of exercise actually supports that and helps [ID12, Leader].*

Whilst most participants asserted the importance of academic attainment, several acknowledged that this was intertwined with health and wellbeing. One head teacher (Principal) noted that the children could be achieving well academically but perceived that, if they were not in good health, their future opportunities would be affected. They noted their school’s context, where a large proportion of children were assessed as vulnerable, so improving health was a prerequisite to achieving the academic performance they aspired to. Using physical activity as a mechanism to improve academic performance was mentioned by another member of leadership staff, noting the need to counteract children’s sedentary lifestyles.

*Obviously I have to decide whether I feel that the intervention is going to be of any benefit to the children, mainly academically because I think that’s our reason to be here but equally for the wellbeing as well and I think the two go hand in hand [ID12, Leader].*

*Obviously academic levels, we’re in a vulnerable school and anything towards that is important…. At the end of the day, they can be as bright as anything but if they can’t get off their sofa because they’re too big then they’ve got real issues [ID9, Leader].*

Several factors that inform prioritisation decisions were mentioned by participants. One issue that was raised frequently was an increase in sedentary lifestyles and physical inactivity. Participants were concerned about children’s future health beyond school and were interested in initiatives that would have long-term impacts on lifestyle behaviours. Many staff spoke about social media and games consoles consuming children’s time and a reduction in outdoor play, sports participation, and active transport for example.

*The way our ethos is, it’s about getting the children healthy, out of this cycle of unhealthiness and obesity which, looking at some of the children already, it’s a major concern what they’re going to be like in four or five years’ time [ID9, Leader].*

*We do feel we’re fighting the tide against social media, and the more we can get our children physically active, the better… We’re becoming a nation of children who live indoors rather than playing outside [ID11, Safeguarding role].*

Some staff spoke about prioritising initiatives that can have an impact within the school day. In some cases, this was in relation to diet, with the participants aware that children may not be responsible for their diet when at home or may choose to independently buy unhealthy foods outside of school. Staff thought that education could only have a limited effect, so providing healthy meals on the premises had the potential to have the most sustainable impact. In addition, engaging in physical activity during the school day could directly impact on energy imbalance that can accumulate when the children are not in attendance.

*As a school, I’d prioritise as much as we could affect…. Obviously, we can’t control all the things that happen outside school. We need to educate them about that and I think that education is what we’re here for and I think we do do that….If you’re doing something regularly—something like The Daily Mile will have a direct impact much more effectively than preaching about what they might eat because I don’t think children have control of that very often [ID12, Leader].*

When discussing potential initiatives that schools may consider implementing, participants referred to government guidance or recommendations for help with identifying the “best” approach. For example, one teacher talked about an initiative to improve children’s wellbeing and how they developed their own school-specific approach that drew on a number of suggested initiatives, as they felt a combined approach was more suitable for their school. Whereas other schools did not feel it necessary to develop a bespoke strategy and were happy to adopt government suggested activities such as “brain breaks”.

*We have done brain breaks and checking in and seeing how everybody is at different points in the day but there isn’t, a government initiative to follow so we’ve just made our own from others’ [ID14, Teacher].*

### 3.2. Evidence

Some schools used data to inform what initiatives to implement. Specifically, two schools reported that their NCMP results did not compare favourably to other local schools, so this motivated them to adopt The Daily Mile to address the issue of excess weight in their school.

*You get the data for obesity and the BMI data. We’re really low, we’re one of the worst schools in Birmingham [ID10, Leader/teacher].*

*I am very conscious of the obesity levels within our children, not just at our school, nationally and wanted to be involved in something to try and change and influence that [ID11, Safeguarding role].*

When discussing what evidence the staff want or need to inform decision making, the most frequently cited was evidence of benefit. In the absence of published evidence of effectiveness, one school evaluated their own initiatives and adapted them in response to their findings. One leadership staff member believed that uptake of initiatives such as The Daily Mile would be enhanced if its benefits could be demonstrated to schools, such as an improvement in academic attainment. Teachers discussed impact in a less quantifiable metric, referring to children’s attitudes to physical activity instead of changes in activity levels and weight.

*This needs promotion because I think lots of schools would be willing to do it if they had the knowledge that it was actually going to be beneficial [ID12, Leader].*

*So we trial it for six weeks and then we look at the end of the six weeks. Has it made the impact? Has it not? If it hasn’t, then we’ll look at different ways to adjust it and change it so it does [ID7, Leader/teacher].*

*I think you’ve got to bring in some enjoyment levels of children because to lead an active lifestyle or healthy lifestyle they’ve got to enjoy some form of exercise. If they don’t enjoy it they’re not going to do it when they’re older [ID1, Teacher].*

The outcomes that staff would hope to observe as a result of implementing public health interventions were discussed. BMI and changes in physical activity were seen as intermediate outcomes, whereas behaviour change and intentions to continue activities outside of school were seen by several staff as outcomes more desirable and indicative of long-term enduring benefit. Outcomes also seen as important other than physical health were wellbeing and mental health. The participants did acknowledge that measuring changes in attitudes and wellbeing were more challenging, yet these would resonate more with decision makers compared to outcomes used in economic evaluations, such as health-related quality of life and BMI.

*I think the economic one is the most difficult one. I think the wellbeing one would make sense to people [ID2, Leader].*

When participants were asked about the consideration of costs of public health initiatives, teachers largely did not think this was their responsibility or felt that it was irrelevant to their position; they referred to leadership staff having responsibility for decision making and budgets. One teacher thought that the cost of an initiative was irrelevant if it was proven to be beneficial. Some staff did refer to time being a cost that was attributable to an intervention. For example, throughout the interviews, the opportunity cost of initiatives was alluded to, particularly in relation to the time spent on academic subjects that would be foregone. One participant discussed the trade-off between the time costs and the benefits The Daily Mile could have for academic attainment, concluding that it was worth it.

*I think if there were proven benefits I don’t think it would matter so much that there was a cost to it because as a school we’re trying things that will improve wellbeing, mental health and, physical health [ID14, Teacher].*

*I think it might have taken some academic time away but actually, the benefits to those academic studies are probably bigger than the time that they’ve lost [ID12, Leader].*

A number of participants recognised the presence of budget constraints, and some drew the link between using budgets and achieving benefit. Several leadership staff expressed a desire for evidence of cost-effectiveness, inadvertently describing what an economic evaluation could demonstrate. They reflected on the need to spend public funds wisely and show that initiatives have observable results to justify implementation. Again, they referred to the time spent doing public health interventions as a cost.

*I think you’d have to really prove its effectiveness and the impact of it because it’s public funds and it’s like anything we do; we, we have to measure impact, and I know this is a time thing, so I suppose, it is a cost [ID2, Leader].*

*So, for example, if we were trying to reduce BMI, and we wanted to pay for somebody to come in and do a sporting activity over a period of time, we would want to see a measurable decrease [ID10, Leader/teacher].*

## 4. Discussion

This study shows that in these schools, leadership staff are the decision makers, with teachers having little direct role in how public health interventions are prioritised. Whilst participants spoke about their desire for evidence of impact and cost-effectiveness to support decision making, the use of formal assessments of intervention effectiveness and economic evaluations were not reported. Where research evidence was used to aid prioritisation, this was generated by the school itself and involved trialling and adapting initiatives. The robustness of these trials was not discussed, and whilst they may address the schools’ immediate and particular needs, it is unlikely to have the rigour of a robust experimental study. Staff did not seem to disregard formal evidence, rather they did not seem aware that it existed. Cost effectiveness analysis seemed particularly useful. While The Daily Mile was largely seen as a “free” initiative, the opportunity cost of time was raised frequently. This was in relation to Maths and English lessons, which contribute to schools’ performance metrics. The outcomes often desired by schools such as wellbeing improvements and behavioural intentions are not typically incorporated into economic evidence [29]. This suggests that current economic evidence does not suit schools’ needs.

Whilst school staff were interested in and saw the value of economic evidence, they had little prior awareness of it. This could be for several reasons, including that it is published in literature they would not usually have access to (e.g., academic journals, largely outside of the education field), reports are not adapted to a school audience so are perceived as inaccessible to non-specialists, or school staff do not have the time to seek out economic evidence. Jessiman et al. [14] identified time and perceived expertise as barriers to using evidence, although they did not explore these issues from a health economics perspective. This issue has previously been identified in healthcare settings but not schools [30]. A perhaps more important issue is that the evidence currently generated is not suited to schools’ needs. Many participants thought that the outcomes typically reported in cost-effectiveness or cost–utility analyses of childhood obesity interventions (e.g., BMI or health related quality of life, respectively) were not thought to be relevant to the school. Rather, outcomes such as wellbeing, behaviour change, and academic attainment were seen as important. These are more aligned with the outcomes upon which schools in the UK are assessed. In addition, the costs mentioned most frequently were opportunity rather than financial costs. Currently the opportunity cost of children’s time is neglected in economic evaluations [31], but it appears that this should be an important consideration from the schools’ perspective. This might also explain why economic evidence does not reach school decision makers, with it seen as unsuitable to inform any changes in practice.

This is the first qualitative study to examine the prioritisation of public health interventions in schools from a health economics perspective. A limitation of this study is the small sample size. Despite repeated efforts, no additional participants could be recruited. Whilst the sample was representative of the intended characteristics, recruiting multiple participants within the same school could have been advantageous. The views of staff that worked in the same environment could have been compared, for example. Some interviews were also relatively short due to the staff not having time to participate and, if they were able, having limited time before they were required back in the classroom. On occasion, they were able to find individuals able to cover classes. The sample might also reflect individuals who are particularly enthusiastic about research and implementing health and wellbeing initiatives in schools. The participants had a broad range of roles and responsibilities, however, and some individuals were notably critical about The Daily Mile. The participating schools were also diverse, reflecting different levels of inspection performance, funding model (local authority maintained or academy funded), urban/rural location, and children’s free school meal eligibility. In relation to inspection performance, location, and funding model, no obvious differences were observed between participating schools and those that declined. The only notable difference was free school meal eligibility, with a larger proportion of deprived schools participating (schools with a higher proportion of children eligible). Whilst these schools would receive a higher allocation of pupil premium funding, which could be used to support health and wellbeing, this was not mentioned by any participants. The study was conducted in the UK, so might not be reflective of international school systems and prioritisation. It does however appear that the difficulties faced by schools in implementing public health interventions are not limited to a UK setting [16]. Despite efforts to broaden the discussion beyond The Daily Mile, alternative findings may have arisen if the interviews had been conducted in the context of a different public health intervention.

Further research could explore the development of school-relevant economic analyses, which would be suited to the decision makers’ needs and be able to inform public health prioritisation decisions. The issue of how best to disseminate to school decision makers could also be explored. This could involve further qualitative interviews, perhaps followed by a Delphi study to build consensus around strategies for improvement. Another issue to be examined is schools’ preferences for local or national level evidence. In the absence of evidence, some schools decided to generate their own, and schools spoke about their schools’ context being an important consideration. Local evidence could be tailored more to their information needs, such as particular outcomes and costs, and be representative of their local context (e.g., level of deprivation, urban or rural location). There may be a paucity of expertise and resource available to generate this evidence in a timely and robust way, however. Collaboration between local government and schools could facilitate this, with the expectation that local government can provide the necessary expertise. Population monitoring of children’s health and wellbeing has been attempted in Australia [32] and Canada [33] using a measure developed for this purpose (the MDI [23]). Routinely collecting such data could allow the impact of local policies on children’s outcomes to be assessed and reported to decision makers to inform priority setting. Alternatively, existing economic analyses could be adapted using local data. This could require fewer resources than bottom-up evidence generation. Appropriate national evidence could already be available (e.g., government reports, published economic evaluations) and be the result of large randomised studies produced by experts in the field. As the suitability of RCTs for public health research is increasingly questioned [34], it could be that small case studies, natural experiments, or economic models that can be adapted are preferred in the future.

## 5. Conclusions

This qualitative study suggests that schools can be an ideal setting to deliver public health interventions, yet school staff may be mindful of the opportunity cost of these interventions in relation to meeting targets in their core academic subjects. Respondents in our study indicated that they would value evidence of both impact and cost-effectiveness to support decision making; however, they demonstrated little awareness of its availability. The study suggests that it is important that economic evaluations align with the decision making perspective to ensure that the costs and outcomes captured are relevant to the context for decision making and the findings are both comprehensible and available. Although some participants acknowledged potential complementarity between the academic and public health objectives, the more general view was that the public health interventions would be supported if there was evidence that these interventions also impacted on academic attainment. Thus, this study has shown that there are clear trade-offs being made within schools between academic attainment and wider wellbeing outcomes. This emphasises the importance of evaluations capturing the value and opportunity costs associated with these interventions from a broad set of perspectives.
